# A Wide QRS Tachycardia in a Patient with Coronary Artery Disease

**DOI:** 10.19102/icrm.2018.090305

**Published:** 2018-03-15

**Authors:** Jeffrey S. Arkles, Andrew E. Epstein

**Affiliations:** ^1^Electrophysiology Section, Division of Cardiovascular Medicine, University of Pennsylvania, Philadelphia, PA, USA

**Keywords:** Atriofascicular pathway, atrioventricular reciprocating tachycardia, wide complex tachycardia

## Abstract

A wide complex tachycardia (WCT) that presents in a patient with a history of coronary artery disease and myocardial infarction is most likely ventricular in origin. We discuss a case of WCT due to a variant of pre-excitation in such a patient.

## Case presentation

**Figure 1:**
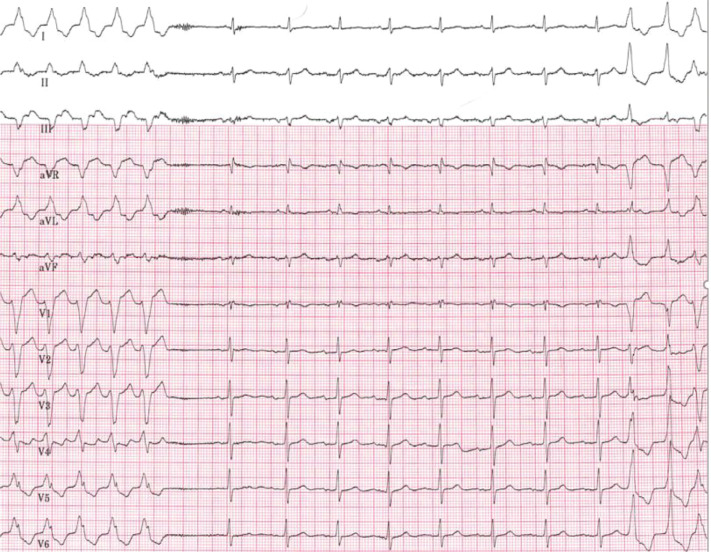
Clinical WCT with termination. 1:1 AV association is present. On the right side of the tracing, a premature atrial beat leads to pre-excitation and initiation of an antidromic tachycardia, as evidenced by the morphology of the last beat on the tracing. The origin of the first and second beats initiating the tachycardia is indeterminate.

A 66-year-old male with a history of coronary artery disease (CAD), prior myocardial infarction as evidenced by an apical scar on nuclear stress test, and a normal left ventricular ejection fraction presented with dizziness and presyncope. His history was notable for supraventricular tachycardia (SVT) and electrophysiology (EP) study three years prior to the current presentation. At the time, atrioventricular (AV) nodal reentrant tachycardia had been diagnosed and a slow pathway ablated; the EP study had been otherwise unremarkable. A wide complex tachycardia (WCT) had then been recorded three years later **(Figure 1)**. Our service was consulted, and the patient underwent a second EP study and ablation procedure.

## Discussion

**Figure 2:**
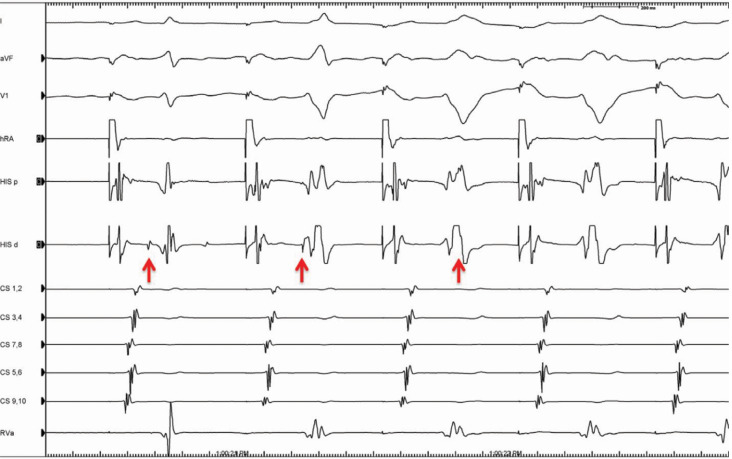
Atrial pacing with prolongation of the PR interval, HV interval shortening, progressive pre-excitation, and a left bundle branch morphology. This is a feature of decrementally conducting accessory pathways, such as an atriofascicular pathway. The red arrows denote the His-bundle potential.

**Figure 3:**
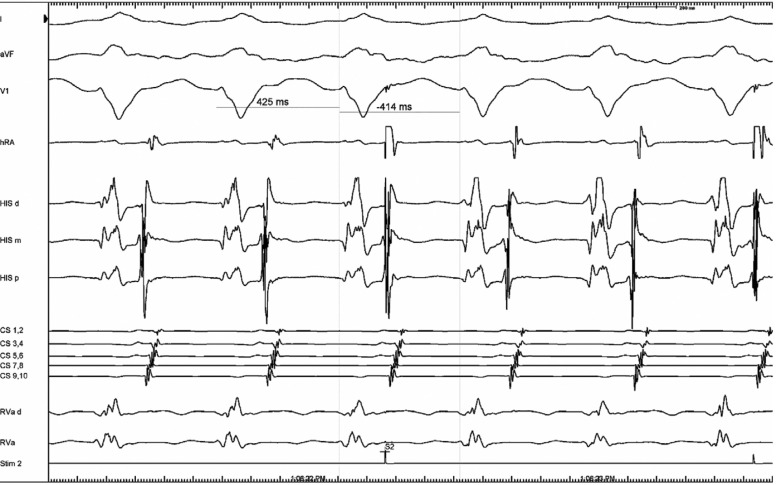
His-bundle synchronous atrial premature beat resetting the tachycardia, proving participation of the accessory pathway tachycardia. Given the decremental pathway conduction, it was difficult to advance the subsequent V-H. Atrial stimuli with greater prematurity would terminate the tachycardia without affecting the septal atrial electrogram.

The EP study was notable for normal baseline intervals, but with right atrial pacing **(Figure 2).** There was prolongation of the PR interval with HV interval shortening, progressive preexcitation, and lengthening of the stimulus-to-pre-excited QRS consistent with an atriofascicular (Mahaim) accessory pathway.^1^ This decremental behavior distinguishes these pathways from typical bypass tracts, which have fixed, non-decremental conduction. Ventriculoatrial (VA) conduction was midline and decremental. A left bundle WCT was easily induced with atrial extrastimuli. Ventricular activation was notable due to a right bundle potential preceding the His-bundle potential. His-bundle refractory atrial premature complexes were delivered and advanced the tachycardia, confirming the diagnosis of antidromic reciprocating tachycardia over a decremental atriofascicular pathway **(Figure 3)**. Ablation was performed on the mid-lateral tricuspid annulus with prompt termination of the tachycardia and loss of antegrade preexcitation.

It was noted that antegrade AV conduction was relatively poor, as AV Wenckebach block occurred at a pacing cycle length of 500 ms, but retrograde conduction was brisk, with VA Wenckebach block occurring at a pacing cycle length of <350 ms. One explanation for the temporal sequence of the two tachycardia presentations is that slow pathway modification damaged anterograde AV nodal conduction, leaving retrograde nodal conduction intact. This allowed for the anterograde-only, decrementally conducting accessory pathway to manifest. Long-term monitoring has not shown any evidence of recurrence.

Left bundle branch block WCTs have an important differential that includes SVT with aberrancy, bundle branch reentry, antidromic reentrant tachycardia, and ventricular tachycardia (VT). The majority of VTs associated with CAD provide a high pretest probability of VT, but careful examination of the 12-lead electrocardiogram suggested another diagnosis, which was confirmed and treated in the EP laboratory.

This case highlights the importance of not assuming that all wide QRS tachycardias in patients with CAD are ventricular in origin, or are due to SVTs with anterograde conduction over the AV node with aberrancy. The tachycardia described herein fulfilled criteria to diagnose VT (specifically, an R-wave in lead V1 of 50 ms, and onset-to-nadir of the QRS being 80 ms in lead V2),^2^ highlighting the caveat that the rules of aberrancy apply when ventricular activation occurs via the normal AV conduction system, and that when the ventricles are activated eccentrically, not via the AV node–His-Purkinje network, that the criteria for VT are fulfilled. Here, rather than treating the patient for VT and implanting a defibrillator, the ablation of an accessory pathway proved curative.

## References

[1] McClelland JH, Wang X, Beckman KJ (1994). Radiofrequency catheter ablation of right atriofascicular (Mahaim) accessory pathways guided by accessory pathway activation potentials. Circulation..

[2] Kindwall KE, Brown J, Josephson ME (1998). Electrocardiographic criteria for ventricular tachycardia in wide complex left bundle brand morphology tachycardias. Am J Cardiol..

